# The usefulness of an application-supported nutritional intervention on non-high-density lipoprotein cholesterol in people with a risk of lifestyle-related diseases

**DOI:** 10.1371/journal.pdig.0000648

**Published:** 2024-12-06

**Authors:** Yuko Noda, Mitsuhiro Kometani, Akihiro Nomura, Masao Noda, Rie Oka, Mayuko Kadono, Takashi Yoneda

**Affiliations:** 1 Department of Health Promotion and Medicine of the Future, Kanazawa University Graduate School of Medical Sciences, Kanazawa, Japan; 2 College of Transdisciplinary Sciences for Innovation, Kanazawa University, Kanazawa, Japan; 3 Department of Cardiovascular Medicine, Kanazawa University Graduate School of Medical Sciences, Kanazawa, Japan; 4 Frontier Institute of Tourism Sciences, Kanazawa University, Kanazawa, Japan; 5 Department of Biomedical Informatics, CureApp Institute, Karuizawa, Japan; 6 Department of Pediatric Otolaryngology, Jichi Medical University, Shimotsuke, Japan; 7 Department of Internal Medicine, Hokuriku Central Hospital, Toyama, Japan; Iran University of Medical Sciences, ISLAMIC REPUBLIC OF IRAN

## Abstract

Lifestyle-related diseases, such as diabetes, are mostly caused by poor lifestyle habits; therefore, modifying these habits is important. In Japan, a system of specific health checkups (SHC) and specific health guidance (SHG) was introduced in 2008. The challenges faced include low retention rates and difficulty in maintaining results. Digital technologies can support self-management and increase patient convenience, although evidence of the usefulness of this technology for SHG is limited. This study evaluated the usefulness of nutritional guidance using a smartphone application (app) added to conventional SHG. We recruited eligible participants for SHG in Japan from November 2018 to March 2020. We assigned them to “Intervention Group: Application-Supported Nutrition Therapy” or “Control Group: Human Nutrition Therapy” based on their desire to use the app. The primary outcome was a change in non-high-density lipoprotein cholesterol (non-HDL-C) levels post-intervention. The secondary outcomes were a change in lipid profile, metabolic indices, and frequency of logins to the app. We assessed 109 participants in two cohorts: 3-month (short-term) and 6-month (long-term). The short-term cohort had 23 intervention and 29 control participants, while the long-term cohort had 35 and 22, respectively. There was a significant improvement in non-HDL-C levels in the short-term intervention group compared to the control group. There was no significant difference in non-HDL-C levels in the long-term groups or at 1 year. There were significant improvements in body weight (BW) in the short-term cohort until 1 year compared within the groups. The retention rate remained high in the short-term cohort (92%) but decreased to 57.8% at 6 months in the long-term cohort. Using an app system to facilitate dietary recordings and guidance for patients at risk of lifestyle-related diseases led to improved lipid levels and BW. These benefits persisted to some extent after 1 year. This app may partially supplement conventional SHG.

## Author summary

Lifestyle-related diseases, including diabetes, are mostly caused by poor lifestyle habits; therefore, modifying these habits is important. In Japan, although a system of specific health guidance (SHG) to modify diet and exercise for people at risk of lifestyle-related diseases has been introduced, the retention rates are low, and maintaining results is difficult. Digital technologies can support self-management and increase patient convenience, but evidence of the usefulness of this technology for SHG is limited. This study evaluated the usefulness of application (app)-supported nutritional intervention. We assigned 109 participants to “Intervention Group: Application-Supported Nutrition Therapy” or “Control Group: Human Nutrition Therapy” and categorized them by intervention period (short-term or long-term cohort). There was a significant improvement in non-HDL-C only in the short-term intervention group. There were significant body weight (BW) improvements in the short-term cohort until 1 year compared within the groups. The retention rate remained high in the short-term cohort (92%) but decreased to 57.8% at 6 months in the long-term cohort. Using an app to facilitate dietary recordings and guidance for patients at risk of lifestyle-related diseases led to improved lipid levels and BW. These benefits persisted to some extent after 1 year. This app may partially supplement conventional SHG.

## Introduction

Lifestyle diseases, such as diabetes and hypertension, mostly stem from habits such as poor diet and lack of exercise. Altering these habits can effectively treat these diseases [1,2]. The collective presence of several obesity-related conditions is called metabolic syndrome [3]. Although intervention for metabolic syndrome tends to be delayed owing to the asymptomatic nature of its early stages, the rate of development of cardiovascular disease is significantly higher in patients with metabolic syndrome, and prevention or early intervention is required [4–7].

In 2008, the Japanese government introduced “specific health checkups (SHCs)” for 40–74-year-old individuals aimed at preventing lifestyle diseases with a focus on metabolic syndrome-related measures, including weight, blood tests (lipid profile and liver function), and blood pressure (BP). Individuals suspected of having metabolic syndrome based on the SHC were provided “specific health guidance (SHG),” such as nutritional guidance for improvement in lifestyle. This guidance was provided by a physician, public health nurse, or nutritionist for a 3- to 6-month period. Although SHCs and SHG were introduced nationwide in 2008, several challenges have been faced, such as a low retention rate and difficulty in maintaining results [8–10].

Although reports suggest that SHG improves the lipid profile and obesity indices [3,9], the attainment rate of guidance was low. Of approximately 5.2 million individuals deemed to require SHG, only 1.2 million (23.2%) successfully completed the guidance process [11]. The dropout rate after the decision to participate was 13–38% (interrupted the first time: 3–12%), and the main reasons for discontinuation included “trying to do it on one’s own” or “being too busy to take the guidance or troublesome to communicate with medical staff” [10–11]. Conventional SHG often requires users to maintain paper-based dietary records, which are labor-intensive and difficult to sustain. In addition, the duration of health guidance and intervention is limited, given limited medical resources; while it is effective during the intervention period, its effectiveness wanes when the intervention ceases [12]. Reducing the patient burden in conventional SHG, improving the guidance effect, and sustaining the effectiveness after the intervention period are important for patients with metabolic syndrome.

Recently, digital technologies have emerged in the medical field to support self-management and increase patient convenience. The usefulness of glycemic control using digital technologies has been reported [13], with some studies reporting higher compliance rates compared to when paper records are used [14]. Conversely, evidence of the effectiveness of digital technologies used in SHG is limited due to the variety of digital technology functions used.

The smart device application (app), “Asken,” is unique in that it has a meal photo analysis system using deep learning, algorithm-based individualized nutritional therapy, and an Internet of Things [IoT]-based biometric data recording system [15,16]. Favorable results regarding the validity of dietary records using Asken have been reported. We hypothesized that incorporating an app capable of precisely managing dietary records, alongside the monitoring feature of standard health guidance, would enhance the convenience and elevate the effectiveness of the intervention. Evaluation of the improvements in efficacy is important when digital technology interventions are used in SHG to understand their usefulness. Therefore, this study aimed to assess the effectiveness of adding smartphone app- and IoT-based nutritional guidance to conventional human-provided SHG in patients with metabolic or pre-metabolic syndrome at risk of lifestyle-related diseases. In terms of outcomes, to evaluate the effects of interventions focused on nutritional guidance, we focused on lipid profiles, particularly non-high-density lipoprotein cholesterol (non-HDL-C) levels, which is a good predictor of ischemic heart disease and can be assessed for several lipid changes, including low-density lipoprotein cholesterol (LDL-C), a factor in atherosclerosis [17,18].

## Results

### Baseline characteristics

**Fig 1:**
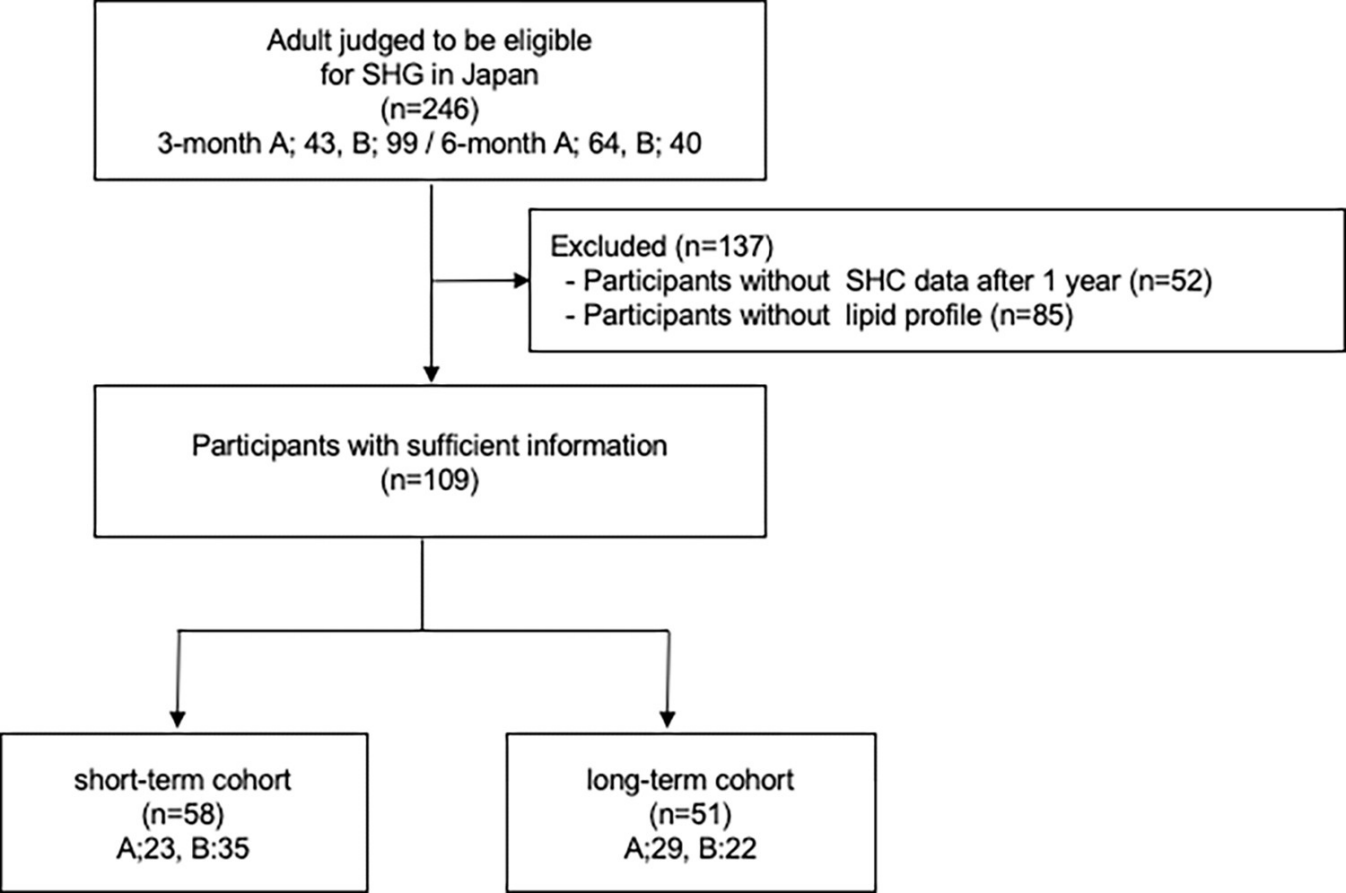
The study flowchart.

We assessed 246 individuals who participated in SHG in Japan from November 2018 to March 2020. We excluded 52 participants without SHC data after 1 year and 85 participants without lipid profile values. The short-term cohort comprised 58 participants, and the 6-month guidance group comprised 51 participants. In the short-term cohort, there were 23 and 35 participants in the intervention and control groups, respectively. In the long-term cohort, there were 29 and 22 participants in the intervention and control groups, respectively (Fig 1).

Table 1 presents the baseline characteristics of the participants. In the short-term cohort, the mean age was 61.25 ± 10.56 years, 34.5% were women, the non-HDL-C level was 143.88 ± 52.44 mg/dl, and body weight (BW) was 70.36 ± 9.09 kg. In the long-term cohort, the mean age was 53.08 ± 6.02 years, 29.4% were women, the non-HDL-C level was 155.35 ± 35.66 mg/dl, and BW was 76.54 ± 10.19 kg.

**Table 1:** Demographic and clinical characteristics of the participants at baseline.

Characteristic	short-term cohort			long-term cohort		
	All n = 58	Intervention n = 23	Control n = 35	All n = 51	Intervention n = 29	Control n = 22
Age, years	61.25±10.56	51.95±8.06	67.09±7.25	53.08±6.02	50.62±5.76	56.32±4.77
Female sex, %	34.5	34.8	34.3	29.4	24.1	36.4
Cholesterol, mg/dl						
Non-high-density lipoprotein	143.88±52.44	158.71±26.10	136.47±60.55	155.35±35.66	160.00±37.25	149.23±33.28
Total cholesterol	204.14±35.37	209.12±26.46	201.50±39.43	209.10±36.49	214.17±36.77	202.41±35.84
High-density lipoprotein	53.90±16.33	53.96±16.89	53.86±16.19	53.75±12.76	54.17±12.91	53.18±12.83
Low-density lipoprotein	126.24±34.46	130.70±22.24	123.31±40.59	133.48±29.40	132.21±30.23	135.24±28.85
Triglyceride, mg/dl	166.34±98.88	173.39±86.40	161.71±107.26	133.73±72.45	151.41±84.86	110.41±43.54
Body weight, kg	70.36±9.09	72.24±7.42	69.13±9.94	76.54±10.19	77.88±10.13	74.77±10.24
Body mass index	25.79±2.35	26.03±2.16	25.63±2.49	27.30±2.38	27.23±2.48	27.40±2.30
HbA1c, %	5.74±0.41	5.82±0.55	5.70±0.29	5.63±0.29	5.64±0.26	5.62±0.33
Blood pressure, mmHg						
Systolic blood pressure	129.81±15.49	128.43±12.32	130.71±17.37	132.22±13.68	128.76±13.03	136.77±13.44
Diastolic blood pressure	79.34±9.53	81.17±9.62	78.14±9.42	83.02±10.35	80.55±10.62	86.27±9.24

Data are presented as mean ± standard deviation. Body mass index is the weight in kilograms divided by the square of the height in meters. Abbreviation: HbA1c, glycated hemoglobin

### Primary outcome

**Fig 2:**
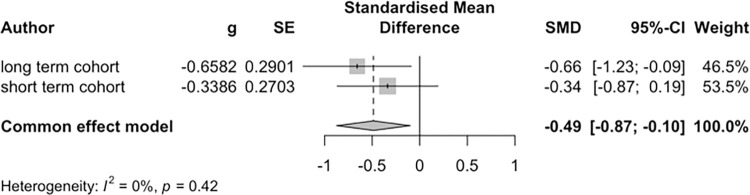
The forest plots of the effects of the intervention on non-high-density lipoprotein cholesterol levels.

The primary outcome was the change in non-HDL-C level from baseline to the end of SHG compared to the control group. Fig 2 shows the forest plots of the intervention’s effects on non-HDL-C levels.

The two cohorts with a total sample of 109 participants were included in the fixed-model meta-analysis for non-HDL-C levels. There was a significant difference between the intervention and control groups (-0.49, 95% CI: -0.87 to -0.10; p < 0.05). The heterogeneity for these studies was not significantly different (I^2^ = 0.0%, p = 0.42) (Fig 2). The mean reduction in non-HDL-C level was -3.97 ± 19.24 mg/dl in the intervention group and 3.78 ± 24.97 mg/dl in the control group for the 3-month guidance group (Table 2).

**Table 2:** ANCOVA results of the impact of the intervention on non-HDL-C level in the 3-month guidance group.

Characteristic	Intervention n = 23	Control n = 35	P-value intervention vs. control
Non-high-density Lipoprotein (mg/dl)	-3.97±19.24	3.78±24.97	0.0162*

Age and sex are considered covariates.

* p < 0.05, intervention group versus control group.

Abbreviation: ANCOVA, analysis of covariance

### Secondary outcomes

#### Three-month SHG group (short-term cohort)

Table 3 shows the lipid profile and metabolic index outcomes within and between the groups, specifically the changes from baseline to after 3 months of intervention and at the 1-year mark. The within-group comparison showed significant improvements in BW and body mass index (BMI) at all time points in both the intervention and control groups. In addition, there was a significant improvement in HDL and TG after intervention in the control group (HDL: 4.54 ± 7.65 mg/dl, p < 0.05; TG: -30.71 ± 83.25 mg/dl, p < 0.05). There were significant improvements in HDL and TG at 1 year in the intervention group (HDL: 4.48 ± 5.63 mg/dl, p < 0.05; TG: -37.00 ± 68.35 mg/dl, p < 0.05). There was a significant worsening in systolic blood pressure (sBP) after intervention in the intervention group (4.30 ± 9.69 mmHg, p < 0.05), and sBP and diastolic blood pressure in the control group (sBP: 12.65 ± 18.45 mmHg, p < 0.05; diastolic BP: 8.47 ± 9.75 mmHg, p < 0.05). In the between-group comparison, there were significant differences in LDL after intervention in the intervention group. There were no significant differences between the groups in other lipid profiles and metabolic indices either after intervention or 1 year later (intervention group: 1.43 ± 18.76 mg/dl vs. control group: 5.43 ± 25.17, p < 0.05).

**Table 3:** Within- and between-group lipid profile and metabolic index changes from baseline to after 3 months of intervention and at the 1-year mark.

	After Intervention (3-month)					After 1 year				
Characteristic	Intervention n = 23	P-value	Control n = 35	P-value	P-value Intervention vs. Control	Intervention n = 23	P-value	Control n = 35	P-value	P-value Intervention vs. Control
Cholesterol, mg/dl										
Total cholesterol	-1.67±4.54	0.7164	8.32±4.51	0.07384	0.167	1.48±3.90	0.7081	-4.85±3.69	0.1972	0.309
High-density Lipoprotein	2.30±1.34	0.09994	4.54±1.29*	0.00127	0.131	4.48±1.17*	0.0009492	0.51±1.10	0.6437	0.1009
Non-high-density Lipoprotein	-3.97±4.01	0.3328	3.78±54.93	0.3771	0.0162**	-3.00±3.78	0.4359	-5.37±3.55	0.1396	0.324
Low-density Lipoprotein	1.43±3.91	0.7172	5.43±4.25	0.2106	0.0266**	2.43±4.02	0.551	-3.17±4.38	0.4741	0.710
Triglyceride, mg/dl	-20.52±21.75	0.3557	-30.71±14.07*	0.03606	0.641	-37.00±14.25*	0.01648	-15.66±13.41	0.2512	0.481
Body weight, kg	-1.83±0.62*	0.007063	-1.94±0.73*	0.01374	0.164	-2.10±0.66*	0.004217	-2.16±0.67*	0.003427	0.566
Body-mass index	-0.70±0.25*	0.00988	-0.52±0.17*	0.004809	0.215	-0.82±0.25*	0.003723	-0.61±0.17*	0.001006	0.732
HbA1c, %	-0.06±0.079	0.4476	-0.03±0.037	0.3733	0.166	0.00±0.083	1	-0.01±0.030	0.8546	0.175
Blood pressure, mmHg										
Systolic blood pressure	4.30±2.02*	0.04462	12.65±3.12*	0.0003401	0.655	2.26±1.76	0.2124	-1.06±2.86	0.7175	0.749
Diastolic blood pressure	3.22±1.99	0.1198	8.47±1.65*	1.517e-05	0.339	1.48±1.41	0.3058	-2.06±1.53	0.195	0.649

Data are presented as mean ± standard error.

* p < 0.05, compared with each group’s baseline.

** p < 0.05, intervention group versus control group.

Abbreviation: HbA1c, glycated hemoglobin

### Six-month SHG group (long-term cohort)

Table 4 shows the lipid profile and metabolic index outcomes within and between the groups and the changes from baseline to after 6 months of intervention and at the 1-year mark. In the within-group comparison, there were significant improvements in HDL, triglycerides (TG), BW, BMI, glycated hemoglobin (HbA1c), and sBP after intervention and sBP at 1 year in the intervention group. There were significant improvements in HbA1c after the intervention and nothing at 1 year in the control group. In the between-group comparison, there were no significant changes at any time point.

**Table 4:** Within- and between-group lipid profile and metabolic index changes from baseline to after 6 months of intervention and at the 1-year mark.

	After Intervention (6 months)					After 1 year				
Characteristic	Intervention n = 29	P-value	Control n = 22	P-value	P-value Intervention vs. Control	Intervention n = 29	P-value	Control n = 22	P-value	P-value Intervention vs. Control
Cholesterol, mg/dl										
Total cholesterol	-4.08±6.47	0.534	26.24±12.87	0.05435	0.658	-9.38±6.72	0.174	22.79±12.50	0.08258	0.219
High-density Lipoprotein	2.66±1.29*	0.04846	4.68±2.36	0.0603	0.543	2.14±1.54	0.1762	2.64±2.02	0.2062	0.935
Non-high-density Lipoprotein	-6.73±5.79	0.2548	21.55±11.70	0.07963	0.410	-11.52±6.57	0.09054	20.15±11.15	0.08489	0.190
Low-density Lipoprotein	-0.79±4.31	0.8553	8.65±9.08	0.3521	0.7493	-6.90±4.94	0.1734	9.73±10.84	0.3798	0.421
Triglyceride, mg/dl	-25.86±10.61*	0.02134	12.00±12.85	0.361	0.943	-19.48±9.77	0.05602	3.68±13.44	0.7867	0.571
Body weight, kg	-1.94±0.81*	0.02298	-0.75±0.70	0.2957	0.1869	-2.03±1.05	0.06333	-1.51±0.74	0.05302	0.767
Body-mass index	-0.68±0.29*	0.02651	-0.27±0.24	0.2788	0.335	-0.66±0.37	0.08772	-0.54±0.29	0.07689	0.908
HbA1c, %	-0.18±0.030*	3.025e-05	-0.14±0.036*	0.004011	0.811	0.00±0.030	1	0.01±0.034	0.7623	0.0765
Blood pressure, mmHg										
Systolic blood pressure	-5.21±2.12*	0.02237	-5.00±3.03	0.1132	0.87119	-5.29±2.20*	0.0255	-3.50±1.80	0.06458	0.269
Diastolic blood pressure	-1.39±1.72	0.432	-3.77±2.62	0.1641	0.707	-2.39±2.11	0.2757	-2.32±1.59	0.1588	0.649

Data are presented as mean ± standard error.

* p < 0.05, compared with each group’s baseline.

** p < 0.05, intervention group versus control group.

Abbreviation: HbA1c, glycated hemoglobin

### Overview of the effects on lipid profile and metabolic indices

Two cohorts (3-month guidance group [short-term cohort] and 6-month guidance group [long-term cohort]) with a total sample of 109 participants were included in the fixed-model meta-analysis for lipid profile and metabolic indices. The results showed significantly better total cholesterol (TC) in the intervention group than in the control groups (-0.51, 95% CI: -0.90 to -0.12; p < 0.05). The heterogeneity for these studies was not significantly different (I^2^ = 0.0%, p = 0.42) (S1 and S2 Figs).

#### Change in the percentage of participants who logged onto the app

**Fig 3:**
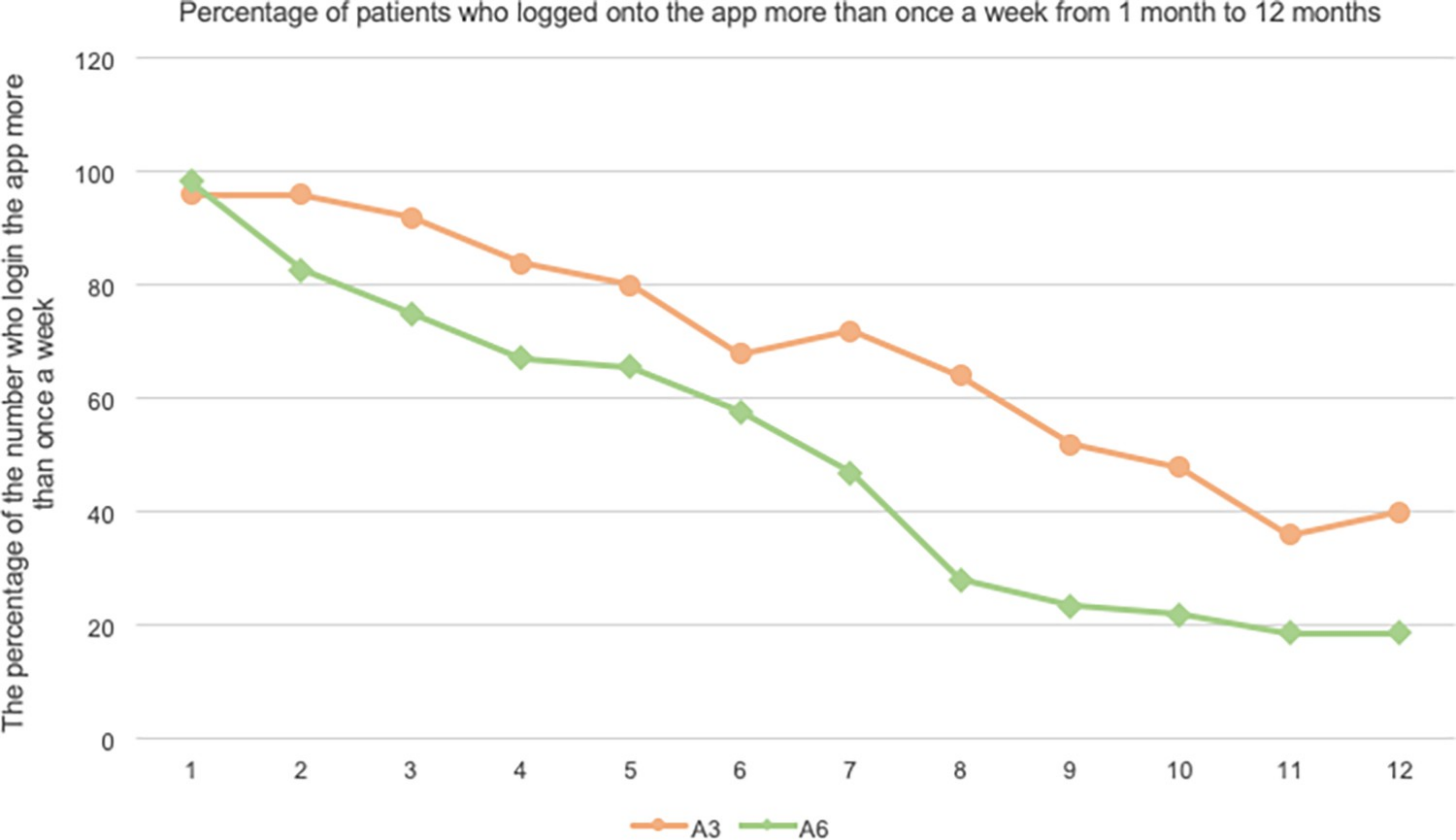
The percentage of participants who logged onto the app more than once a week from 1 to 12 months.

Fig 3 shows the percentage of participants who logged onto the app more than once per week for 1 month to 1 year. The percentage of those who logged onto the app at least once a week decreased to 92% at the end of the guidance (3 months) and to 40% at the end of 1 year in the 3-month guidance group. In contrast, in the 6-month guidance group, 57.8% logged onto the app at the end of the guidance (6 months) and 18.8% at the end of 1 year. There was a significant decrease in this rate in the 6-month SHG group (p < 0.05).

## Discussion

### Principal results

This study aimed to evaluate the efficacy of nutritional guidance delivered by app and IoT systems using a smartphone app combined with conventional SHG provided by a nutritionist in patients with metabolic syndrome at risk of lifestyle-related diseases. Several important findings were obtained in this study. First, we found that there were significant improvements in non-HDL-C levels after intervention in the intervention group when compared with the control group in the 3-month guidance group. Second, in the within-group comparison, more indicators tended to improve significantly in the intervention group, especially in the 6-month guidance group. Third, the retention rate remained high in the 3-month guidance group (92%), whereas there was a significant decrease in the retention rate in the 6-month guidance group.

### Comparison with prior work

First, in terms of the primary outcome measure, non-HDL-C level, there was a significant improvement in the 3-month guidance group. These results most likely occurred due to the addition of the app to conventional SHG. In terms of the amount of change, the intervention group improved by -3.97 ± 19.24 mg/dL, while the control group increased by 3.78 ± 24.97 mg/dL. A previous study reported a change in non-HDL-C levels of -8.6 mg/dL in the 3-month intervention group using a mobile app and 0.6 mg/dL in the control group [19], and these values did not differ greatly from ours. In addition to SHG, the intervention method in this previous study used biometric information measured by an advanced BP monitor, blood glucose meter, pedometer, and other devices through an app for 3 months, and feedback on this information was received. Additionally, participants were asked to measure blood glucose and blood pressure twice a day. In this study, in contrast to significant reductions in BW and BMI, all remaining physical and metabolic parameters, including BP, cholesterol, TG, and HbA1c, showed no difference between the intervention and control groups. This study differed from the present study in that their participants were all men, the specific health instructions were provided in a group session, the app was different, and participants were asked to test items such as blood pressure and blood glucose more frequently compared to our study. In terms of differences in non-HDL-C level changes, previous studies have reported that men are more likely to be robust users [20], frequent self-measurement and recording are effective in weight loss programs [21], and there is a correlation between weight loss and non-HDL-C levels [22]. Therefore, these aspects may have influenced the results.

Second, in terms of secondary outcomes, within-group comparisons showed a trend toward significant improvement in more measures in the intervention group than in the control group, especially in the 6-month guidance group. In the 3-month guidance group, improvements were observed for BW and BMI in the intervention group and for HDL, TG, BW, and BMI in the control group. In the 6-month guidance group, improvements were observed for HDL, TG, BW, BMI, HbA1c, and sBP in the intervention group and HbA1c in the control group. At the 1-year follow-up, in the 3-month guidance group, improvements were observed for HDL, TG, BW, and BMI in the intervention group and for BW and BMI in the control group. In the 6-month guidance group, improvements were observed for sBP in the intervention group, and there were no improvements observed in the control group.

According to previous studies, the amount of change in BW after 1 year of SHG was -0.29 kg (Fukuma) to -2.25 kg (Tsushita) [9,23]. Compared to data from the 1-year follow-up in the control group (SHG only) in this study, the amount of weight change was within the range reported above, indicating that average health guidance was provided. Although, in the 6-month guidance group, at 1 year, neither group showed a significant change in weight (after intervention [control]: -0.75 kg; 1 year [intervention]: -2.03 kg; [control]: -1.51 kg). These results also suggest that the app-using group might have been universally successful in losing weight, regardless of the duration of the guidance period. Other studies using mobile apps have reported weight changes of -3.0 kg after 3 months of intervention but have also reported difficulties in maintaining the effect after the end of the intervention (Kondo) [19]. In this study, the participants were permitted to continue using the app voluntarily even after the end of the health guidance, which might have played a role in continuing to assist them with their lifestyle habits, which they now had to control solely on their own. In the 3-month guidance group, although HDL and TG were significantly different in the control group after the intervention, the differences were no longer significant after 1 year; instead, a significant difference was observed in the intervention group after 1 year. As a general trend, it was easy to see the effect immediately after the intervention and difficult to maintain the effect over time. It was interesting to note that the number of indicators that showed significant improvement within groups decreased over time for the control group in the 3-month guidance group and both the intervention group and the control group in the 6-month guidance group. In contrast, the number of indicators that showed significant improvement increased only for the intervention group in the 3-month guidance group. The results suggested that although a 3-month period is not enough time to see the maximal effect of the added app intervention when human guidance was completed over a short period, the effect of app intervention gradually emerged, sustaining and strengthening lifestyle improvements from later periods up to 1 year later. Conversely, the add-on effect of the app was maximized and gradually decreased as app usage declined when the app was used in conjunction with long-term human guidance (6 months).

Third, the number of participants who logged onto the app at least once a week remained high at 92% in the 3-month guidance group, but in the 6-month guidance group, it gradually declined to 57.8% and further declined throughout the year to almost 20%. In a previous study by Patel et al. involving a 12-week intervention using an app for weight and diet management, the retention rate was 87.5% at 1 month, 75% at 3 months, and 78% at 6 months [24]. Withdrawal rates in various apps vary but are reported to be roughly 10–40% [25]. The 3-month retention rate was considered reasonable in comparison to previous studies. In a previous study by Okaniwa et al., the combination of the Asken app with human-delivered video messaging (intervention group) resulted in a significantly lower dropout rate from the program compared to using the app for food recording and text messaging alone (control group). The Cox proportional-hazards model estimate showed a hazard ratio of 0.078, statistically significant at the 5% level [26]. Although this study does not include video messaging, the human intervention suggests a favorable environment for continued app use. During the follow-up period, app use after completing SHG was voluntary, and the retention rate gradually declined, which was expected. Despite the decreased frequency of app use, improvements in several lipid parameters were sustained in the intervention group after 1 year. In addition, reports suggest that the higher the level of involvement with the app, the greater the reduction in weight loss and HbA1c [27–29]. These results suggest that efforts to increase the frequency of app use would further enhance the improvement effect. However, there are also reports that when the requirement for recorded data is too high, this is burdensome for users [30]; therefore, it is necessary to examine the balance between the amount of intervention and its effects. Regarding recruitment, some participants who had previously refused to participate in SHG decided to do so after using the app, suggesting the possibility of increasing the uptake of SHG through a variety of avenues. Further research is needed to determine the factors that influence the frequency of use and persistence rate of participants.

### Strengths and limitations

The strength of this study was that the effects of the app, as an addition to the SHG currently provided in Japan, were analyzed for each period of the intervention. Furthermore, by observing the progress of the study up to 1 year later, we were able to gain insight into the long-term effects of the intervention. Despite these strengths, the study had several limitations. First, because it was an open-label design, patients in the intervention group knew they were receiving an empirical psychiatric intervention, and as a result, the intervention may have had better outcomes than the control group. Furthermore, self-selection bias may have been present in this study because the choice to use the app was left to the individual. Second, because the app was added to conventional nutritional guidance, it was difficult to determine the effectiveness of the app by itself. In this regard, the demonstration took the form of conventional guidance versus app only, as not providing conventional nutritional guidance would be ethically problematic. One option considered was to conduct a crossover study to account for confounding factors, but it should be kept in mind that a history of SHG has been reported to influence the effectiveness of the guidance [31].

In conclusion, the addition of dietary recording and nutritional guidance delivered by app and IoT systems for patients with metabolic syndrome and at risk of lifestyle-related diseases showed some improvement in lipid levels and BW. The effect was maintained to some extent 1 year later. This app may be useful for supplementing and following up on conventional SHG. However, further research is needed to identify the most efficient intervention method, as the effectiveness of the app itself depends on the retention rate.

## Materials and Methods

### Study design

This was a prospective, multicenter, unblinded, parallel study to compare the efficacy of nutritional guidance delivered via smartphone apps and IoT systems added to conventional SHG in patients with metabolic syndrome or pre-metabolic syndrome at risk of lifestyle-related diseases. We divided the SHG subjects into two groups (“Intervention Group: Application-Supported Nutrition Therapy” and “Control Group: Human Nutrition Therapy”). We evaluated the changes in non-HDL-C levels as the primary outcome from baseline until 3 months at the end of SHG. The Institutional Review Board of Kanazawa University approved the study protocol on April 13, 2018 (IRB no. 2623–3); written informed consent was obtained from all patients. This trial was registered in the UMIN-CTR (University Hospital Medical Information Network) (https://www.umin.ac.jp/, UMIN ID: UMIN000034751) and was conducted in compliance with the Declaration of Helsinki. Patient recruitment started on November 2, 2018, and was scheduled to continue until March 31, 2023. Data were collected from the following locations: Ishikawa Health Service Association, Kanazawa City Welfare Health Center, Hokuriku Central Hospital, Saiseikai Kanazawa Hospital, Hokuriku Electric Power Company Clinic, and Houju Memorial Hospital. A webpage addresses where interested researchers can apply for access as follows. https://k-slim.w3.kanazawa-u.ac.jp/

### Overview

**Fig 4:**
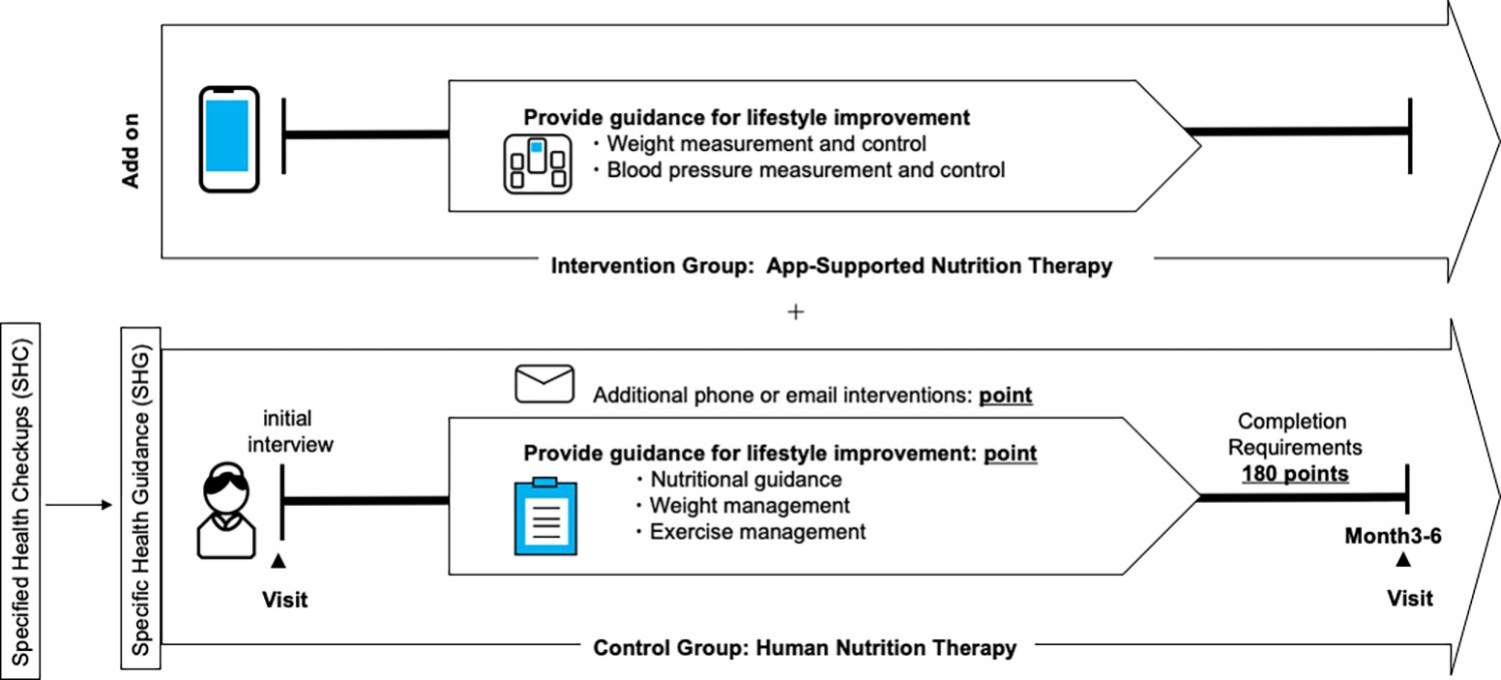
Overview of the schedule of the intervention and control groups. After determining whether health guidance is necessary based on the results of the specific health checkups, the participants are enrolled in either the “Intervention Group: application-supported Nutrition Therapy” or “Control Group: Human Nutrition Therapy” based on their consent to use the app. After the requirements of the SHG are complete, the effects of the intervention are assessed.

**Fig 5:**
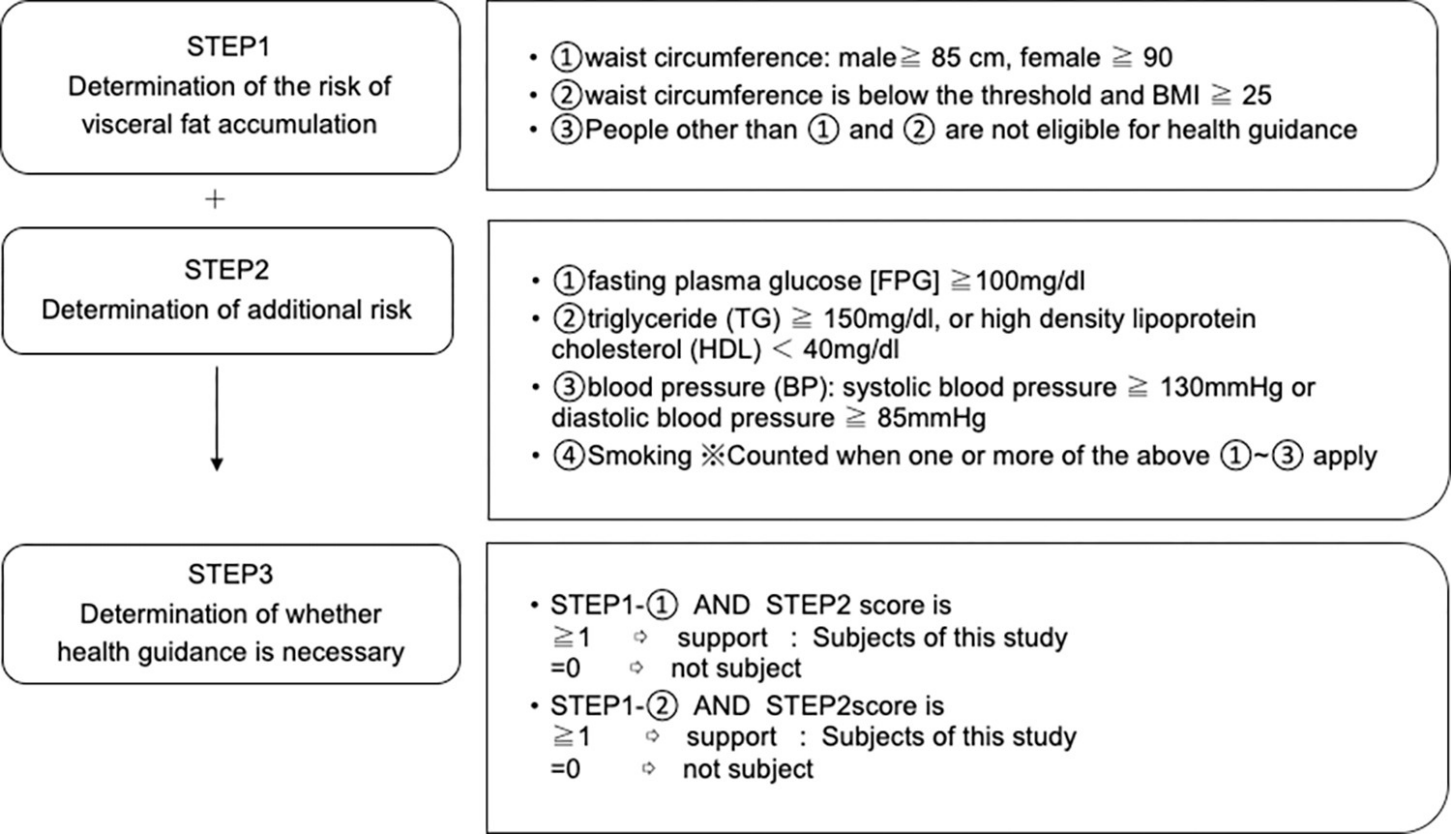
Criteria for Specific Health Guidance (SHG). Using items concerning the risks of visceral fat accumulation and additional risks, the need for SHG is determined.

Fig 4 provides an overview of the schedule for enrollment, interventions, and assessments. Fig 5 shows the selection criteria for SHG based on the results of the SHCs. After determining that health guidance was necessary, participants were briefed on the study after the initial interview. Individuals who agreed to participate by using the smartphone app and to record their weight on the app at least twice in the first 2 weeks were included in the Intervention Group: Application-Supported Nutrition Therapy. Individuals who did not agree to use the app but agreed to allow us to use information such as blood test results were included in the Control Group: Human Nutrition Therapy.

The basic guidance sequence involved an initial interview session, conducted individually or in a group setting, facilitated by a public health nurse or nutritionist. During this session, participants contemplated their lifestyle choices, established behavioral objectives, and subsequently underwent an assessment of outcomes following a designated intervention period. Traditionally, SHG was implemented over 6 months. However, after a review of the process by the Ministry of Health and Welfare (MHLW) in 2017, evaluation after 3 months is also now undertaken, so the duration of the guidance varied depending on the facility: 3 months, 6 months, or both. For this reason, we divided the survey into two groups according to the period: “facilities implementing the plan for 6 months” and “facilities implementing the plan for approximately 3 months.” Each intervention was assigned a specific point value, contingent on its content and duration. A predetermined point threshold (180 points) was a requirement for the completion of the intervention. This approach guaranteed consistent intervention intensity, irrespective of the varying durations. In Japan, the cost of SHG and SHCs is borne by health unions and other organizations; therefore, there is no individual burden.

### Participants

We selected participants who were judged eligible for SHG at hospitals or clinics belonging to the Ishikawa Medical Association in Japan between November 2018 and March 2020. The participants were recruited mostly via self-selection. Some became aware of the program through individual recommendations from public health nurses at the SHCs. Advertisements through flyers and posters distributed at hospitals were also used to recruit participants. The participating hospitals and clinics were Ishikawa Health Service Association, Kanazawa City Welfare Health Center, Hokuriku Central Hospital, Saiseikai Kanazawa Hospital, Hokuriku Electric Power Company Clinic, and Houju Memorial Hospital. The Ishikawa Health Service Association, Kanazawa City Welfare Health Center, and Saiseikai Kanazawa Hospital adopted a 3-month guidance plan. The Hokuriku Central Hospital Hokuriku Electric Power Company’s Clinic and Houju Memorial Hospital adopted a 6-month guidance plan.

The inclusion criteria were: (1) an age 40–75 years (regardless of sex), (2) receiving SHG, (3) provision of written consent for participation in the study, (4) ability to operate a smartphone (evaluation by questions), and (5) ability to record the measurements on the app (recorded at least once per week continuously for 2 weeks after the initial guidance). The exclusion criteria were: (1) not eligible for SHC; (2) under medical treatment for hypertension, dyslipidemia, or diabetes; and (3) determined to be inappropriate as a research subject by the principal investigator or sub-investigator.

### Outcomes

The primary outcome in the 3-month guidance group was a change in non-HDL-C levels from baseline to the end of SHG compared to the control group. Lipid profiles are important indicators of the effectiveness of lifestyle interventions and for the primary prevention of cardiovascular disease in younger people [19,20]. We assessed LDL-C, which is a known risk factor for atherosclerosis, and non-HDL-C, which is the preferable indicator of cholesterol in the Japanese population, according to a national review [17]. Non-HDL-C is less influenced by the immediately preceding meal and considers other atherosclerosis risk factors, such as TG-rich lipoproteins, including very LDL and intermediate-density lipoprotein [21,22]. People with metabolic syndrome and diabetes tend to have high TG levels, and high TG also raises TG-rich lipoproteins [32]. Since LDL alone does not account for these values, non-HDL-C level was set as the primary outcome to compare the amount of change between the groups in this study.

Secondary outcomes were assessed according to the guidance period (3 months or 6 months). These were: (1) amount of change in lipid profile (TC, TG, HDL-C, non-HDL-C, and LDL-C) compared within and between groups; (2) metabolic indices (BW, BMI, HbA1c, and blood pressure) compared within and between groups; and (3) recorded data on the Asken app (login frequency) depending on the period of SHG.

### Intervention group: app-supported nutrition therapy, Asken

Asken is one of the most popular Japanese healthcare apps that supports diet and behavioral change. It has over seven million users. The app comprises several elements as follows: 1) food recording and automatic photo analysis using deep learning, 2) algorithm-based nutrition therapy, 3) biometric data recording system, and 4) other functions.

**Fig 6:**
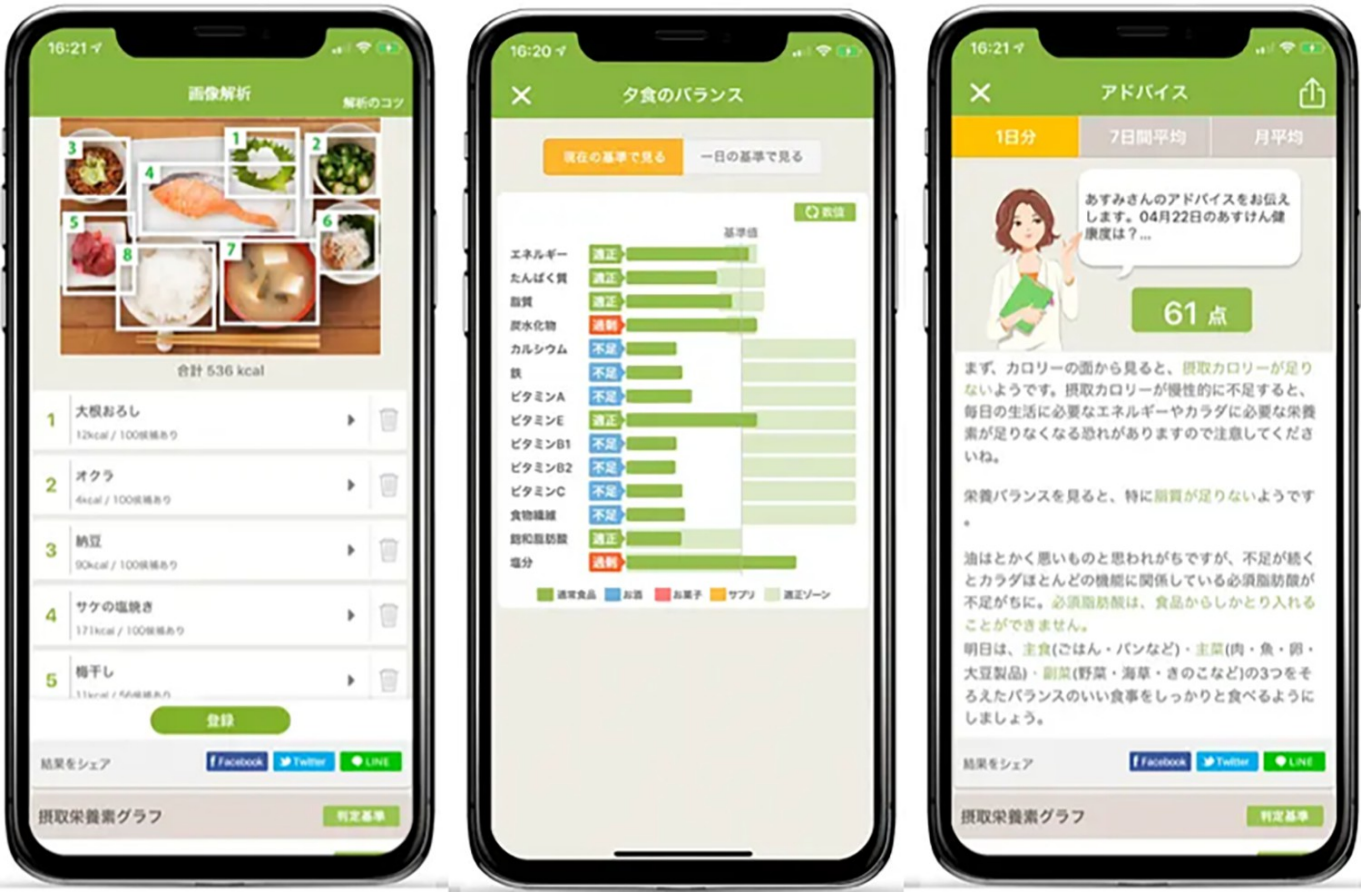
Screen-shot of the automatic photo analysis using deep learning, daily / per-meal intake graph by each nutrient, and a food content rating out of 100.

The food recording and automatic photo analysis is an automatic photo analysis system using deep learning linked to an extensive database of over 100,000 menus, both on the market and homemade. Deep learning has enabled a significant increase in the types of dishes that can be automatically identified and has improved identification accuracy. When users take a picture of their whole meal, the system instantly identifies each item’s frame, menu, and serving amount (Fig 6). Detailed and accurate data can be obtained by selecting an accurate type or brand from the database. Furthermore, if a meal has leftovers, it is possible to adjust the data by recording a half-serving. Asken calculated the energy and nutritional values based on the “Standard Tables of Food Composition in Japan 2015, seventh revised edition” [33]. The validity and accuracy of Asken’s estimated total energy intake and macronutrients, such as carbohydrates, fat, and protein, were demonstrated in an earlier study [16]. Following each food log, Asken shows how well the user has satisfied their recommended range of daily intake of each nutrient with a graph (Fig 6). Second, the algorithm-based nutrition therapy was determined based on the Dietary Balance Guide formulated by the Ministry of Health, Labour, and Welfare and the Ministry of Agriculture, Forestry, and Fisheries. Based on the data obtained from the dietary analysis, personalized nutritional guidance advice messages were displayed. All nutritional guidance was supervised by a nutritionist. The nutritionist, "Miki," selected and commented on the best messages from the pool, ensuring that no unusual or extreme dietary recommendations were made. As the entire process, from menu identification with photo analysis to the generation of dietary feedback, is fully automated, patients can obtain detailed nutritional feedback anywhere and anytime. These dietary messages and feedback were also delivered by the female Japanese character, Miki; there are more than 200,000 different patterns for her wording and facial expressions. Miki always remains compassionate and empathetic toward the patients, even when they eat too much or skip a food intake recording. Miki’s costume is changed with each seasonal event, and greenery backgrounds are provided to keep the app fun and interesting (Fig 6). Third, the biometric data recording system can be used optionally. The recorded BW and footsteps are represented by a graph for easy analysis, and it is possible to record data automatically using Bluetooth or a data-sharing function with other apps. Fourth, there are peer support systems, which comprise a community with the same diet goals and an in-app diary function.

Participants were asked to log onto the Asken app at least once weekly to enter their meals and snacks and receive advice.

Specific Health Guidance is delivered by a female Japanese character, “Miki.”

### Control group: human nutrition therapy

The principle of nutritional therapy in the control group was the same as that in the intervention group, namely, total caloric restriction with macronutrient distribution, as recommended by the MHLW. At baseline, public health nurses/nutritionists conducted approximately 20 minutes of individual or group face-to-face interviews to estimate current energy and nutrient intake. They worked with the participants to develop actionable strategies to improve their dietary habits. The participants received printed materials to describe their plans and record their meals. MHLW-certified public health nurses and nutritionists conducted SHG according to set guidelines. For guidance, abnormal lipid profile values and metabolic indices were considered. For BW, the lower limit of the BMI target was 18.5 kg/m^2^ for those aged 18–49 years, 20.0 kg/m^2^ for those aged 50–69 years, and 21.5 kg/m^2^ for those aged 70 years or older, and the target BW was set individually after confirming the risk factors for brain and cardiovascular diseases [34].

### Data collection

At SHCs, after an overnight fast, venous blood samples were drawn from the antecubital vein by a nurse. Subsequently, the samples were transported to each hospital’s central laboratory or specific clinical specimen examination facilities for analysis. The lipid profile was measured using enzymatic analytical chemistry, and plasma glucose levels were assessed using the glucose oxidase method. HbA1c was measured using high-performance liquid chromatography. Resting blood pressure was measured in the sitting position using an automatic device after 5 min of rest. BMI and waist circumference were measured according to previously published methods.

### Statistical analysis

We defined the 3-month guidance group as a short-term cohort and the 6-month guidance group as a long-term cohort. Patient characteristics and baseline lipid profiles are presented using descriptive statistics, including mean and standard deviation for continuous variables and proportions for categorical variables. For continuous variables, such as lipid levels, the mean and standard deviation of the change before and after intervention were calculated using an analysis of covariance adjusted for age and sex, and groups were compared. A paired *t*-test was used to compare changes within the groups. Data are expressed as mean ± standard error. In addition, the two cohorts (short-term and long-term) with a total sample of 109 participants were included in the fixed-model meta-analysis for non-HDL-C and other indicators. All analyses were conducted using the R software version 3.6.3 (R Foundation for Statistical Computing, Vienna, Austria). Statistical significance was set at a p-value of less than 0.05.

## Supporting information

S1 Fig The forest plots of the effects of the intervention on TC, HDL, LDL, and TG levels. (TIFF)

S2 Fig The forest plots of the effects of the intervention on BW, BMI, HbA1c, sBP, and dBP. (TIFF)
