# Supplementary figures and images for: The usefulness of an application-supported nutritional intervention on non-high-density lipoprotein cholesterol in people with a risk of lifestyle-related diseases
Source: PLOS Digit Health. 2024 Dec 6;3(12):e0000648. doi: 10.1371/journal.pdig.0000648 (PMC11623450; doi:10.1371/journal.pdig.0000648)

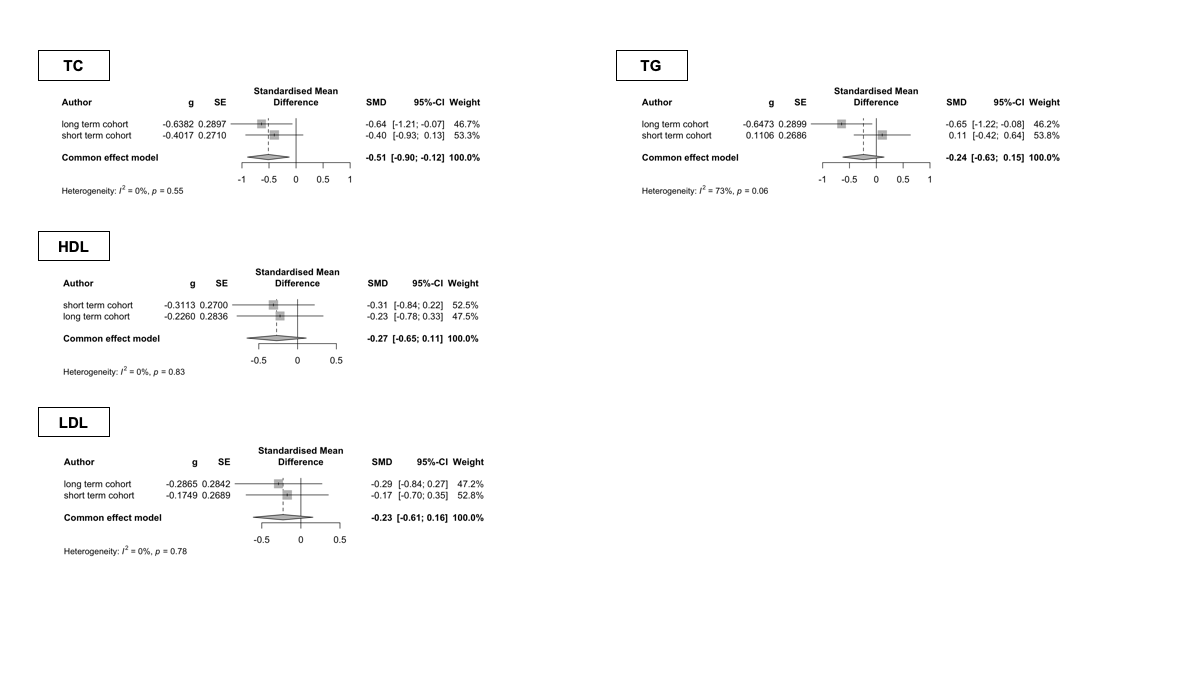

Supplement: S1 Fig — (TIFF) [file pdig.0000648.s001.tiff]

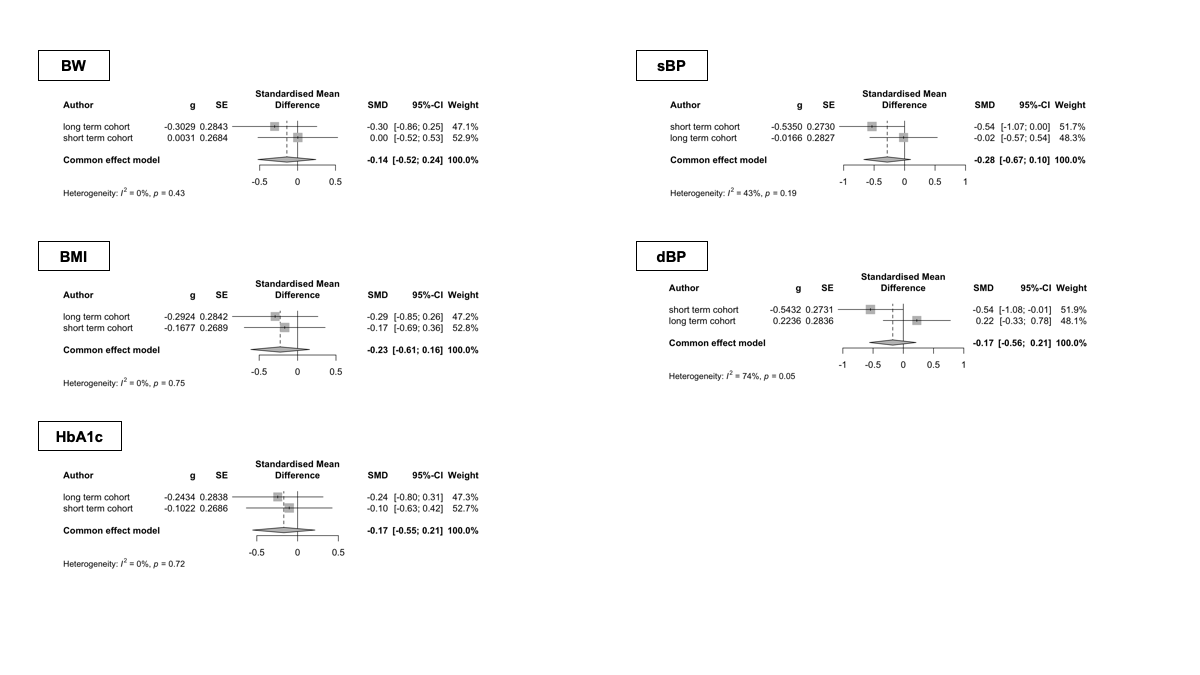

Supplement: S2 Fig — (TIFF) [file pdig.0000648.s002.tiff]
